# Clinical and laboratory factors associated with scrub typhus and hemorrhagic fever with renal syndrome in Southwestern Korea: A cross-sectional study

**DOI:** 10.1371/journal.pntd.0013709

**Published:** 2025-11-07

**Authors:** Thinley Dorji, Choon-Mee Kim, You Mi Lee, Jun-Won Seo, Da Young Kim, Na-Ra Yun, Kwang Jun Lee, Dong-Min Kim

**Affiliations:** 1 Department of Internal Medicine, Central Regional Referral Hospital, Gelephu, Bhutan; 2 Department of Internal Medicine, College of Medicine, Chosun University, Gwangju, Republic of Korea; 3 Premedical Science, College of Medicine, Chosun University, Gwangju, Republic of Korea; 4 Division of Zoonotic and Vector Borne Diseases Research, Center for Infectious Diseases Research, Cheongju, Republic of Korea; University of Connecticut College of Agriculture Health and Natural Resources, UNITED STATES OF AMERICA

## Abstract

**Background:**

Scrub typhus and Hemorrhagic fever with renal syndrome (HFRS) are prevalent infectious diseases in South Korea. This study compared the clinical and laboratory characteristics associated with scrub typhus and HFRS treated at Chosun University Hospital, South Korea.

**Method/Findings:**

A retrospective cross-sectional study was conducted by reviewing the medical records of patients diagnosed with scrub typhus and HFRS between 2010 and 2023. Diagnoses were confirmed through molecular and serological testing. A total of 156 patients with scrub typhus and 45 patients with HFRS were included. Among patients with scrub typhus, 136 (87.18%) patients had Boryong strain, 4 (2.56%) had Taguchi strain, 3 (1.93%) had Karp strain, and 1 (0.64%) had Kanda strain. Most cases of scrub typhus and HFRS occurred in individuals aged ≥65 years. The common clinical features of scrub typhus included fever, fatigue, skin rash, and eschar. In contrast, HFRS commonly presented with fever, gastrointestinal symptoms, and hemorrhagic manifestations. The mean hospital stay was significantly longer for HFRS (14.09 ± 7.67 days) compared to scrub typhus (7.89 ± 7.56 days, *p* < 0.001). A higher proportion of patients with HFRS (15, 33.33%) required intensive care unit admission compared to patients with scrub typhus (14, 8.97%, *p* < 0.001). Among patients with scrub typhus, the adjusted odds of presenting with a skin rash, eschar, or lymphopenia (<1500/µL) were 1.37 (95% confidence interval [CI]: 1.18–1.59, *p* < 0.001), 1.25 (95% CI: 1.11–1.40, *p* < 0.001), and 1.83 (95% CI: 1.34–2.49, *p* < 0.001), respectively.

**Conclusions:**

While scrub typhus and HFRS share overlapping clinical features, skin rash, eschar, and lymphopenia were more commonly associated with scrub typhus. Further studies are warranted to characterize clinical features and outcomes of various hantavirus subtypes.

## Author summary

Scrub typhus and Hemorrhagic fever with renal syndrome (HFRS) both present as acute febrile illnesses with overlapping clinical features. This study describes cases of scrub typhus and HFRS that were confirmed through molecular and/or serological testing at the Chosun University Hospital in South Korea. Data from 201 patients – 156 with scrub typhus and 45 with HFRS are presented in this study. The common symptoms of scrub typhus included fever, fatigue, skin rash, gastrointestinal symptoms, and myalgia. Eschar was documented in three-fourths of patients with scrub typhus. The common symptoms of HFRS were fever, gastrointestinal symptoms, myalgia, and hemorrhagic manifestations. The mean duration of hospitalization was significantly shorter for patients with scrub typhus compared to patients with HFRS. The mortality rate was low in both groups. Patients with a skin rash, eschar, and lymphopenia are more likely to have scrub typhus than HFRS. Such comparisons of clinical and laboratory parameters would help guide clinicians in their diagnostic work up and in case management.

## Introduction

Scrub typhus is a leading cause of acute febrile illness in the Asia-Pacific region, with a pooled seroprevalence of ~25%, and up to ~60% of individuals showing evidence of prior infection (IgG positivity) [1]. Scrub typhus is caused by *Orientia tsutsugamushi*, an obligate intracellular bacterium transmitted through the bites of infected chigger mites [2]. While scrub typhus is reported across many countries, the highest burden occurs within the “tsutsugamushi triangle” [3]. Global mortality rates range from 1.4% among treated patients to 6% among untreated individuals [3].

South Korea has been reporting a steady increase in the cases of scrub typhus after it has been designated as a notifiable disease by the Korea Diseases Control and Prevention Agency. Between 2001 and 2013, there were 70,914 cases reported [4]. The overall annual incidence rate of scrub typhus was 12.2 cases per 100,000 residents for the period 2008–2012 [5]. The trend of cases reported has continued to increase with risk factors such as outdoor activities [6], deforestation [7] and climatic factors such as temperature and precipitation [8].

Hantavirus infection is another significant cause of acute febrile illness, with a substantial caseload reported from the Asia-Pacific region [9]. Hantaviruses are zoonotic viruses, with rodents serving as their primary natural reservoirs. These viruses belong to the genus *Orthohantavirus*, family *Hantaviridae*, and order *Bunyavirales*. Common hantavirus variants reported in Asia include Hantaan virus (HTNV), Puumala virus (PUUV), Seoul virus (SEOV), Muju virus (MUJV), and Soochong virus (SOOV) [9]. Hantaviruses cause two major clinical syndromes: hemorrhagic fever with renal syndrome (HFRS) and hantavirus cardiopulmonary syndrome (HCPS). The global seroprevalence of hantavirus infections is 2.95%, with a higher prevalence of 6.84% reported in Asia [10]. Although mortality rates can reach 15% in some studies [10,11], global data on variant- or syndrome-specific mortality rates remain limited.

Hantaviruses are endemic to several Asian countries, such as China, South Korea and Japan [12]. Hantavirus was first identified in a soldier stationed in the center front, known as the Iron Triangle [13]. Between 2001 and 2017, there were 7048 cases of HFRS and 31 deaths reported in South Korea with an annual incidence of 0.83 per 100,000 residents and a case fatality rate of 1.26% [14]. The majority of HFRS cases occur during seasonal peak noted between October and December [13].

Scrub typhus and hantavirus infections share overlapping clinical presentations, including acute febrile illness and thrombocytopenia, and they are endemic in similar geographic regions. HFRS progresses through five clinical stages: febrile, hypotensive, oliguric, diuretic, and convalescent phases [9]. Similarly, severe scrub typhus can present with organ dysfunction, including shock, acute kidney injury, respiratory failure, and multiorgan dysfunction [15]. This study compares the clinical, laboratory, and epidemiological characteristics of scrub typhus and HFRS, using molecular and serological diagnostic confirmation, in patients treated at a tertiary-care teaching hospital in South Korea.

## Methods

### Ethics statement

This study was approved by the Institutional Review Board of Chosun University Hospital (IRB No. 2025-06-017), Gwangju, Republic of Korea. Informed written consent was waived of as this was a retrospective review of records. No patient identifiers were collected for this study.

### Study design and setting

This retrospective cross-sectional study reviewed data from patients diagnosed with scrub typhus and HFRS who were treated at Chosun University Hospital, a tertiary-care facility in Gwangju, Republic of Korea, between 2010 and 2023. This hospital caters to the population of the metropolis and also to cases referred from smaller provincial hospitals.

There was no standardized protocol for diagnostic testing of febrile illnesses at the study site. Testing for specific pathogens such as *Orientia tsutsugamushi* or hantaviruses was performed at the discretion of the attending physician, based on clinical suspicion derived from patient history, physical examination, and baseline laboratory results.

### Data variables and data collection

Data were extracted from the hospital’s electronic medical records and included the following variables: sociodemographic data (age, sex, occupation, place of residence, and month of hospitalization), clinical data (symptoms and signs at presentation), hospital course (duration of hospital stay, ICU admission, and hospitalization outcomes), and laboratory parameters.

#### Diagnosis of scrub typhus. 

The diagnosis of scrub typhus was confirmed using nested polymerase chain reaction (nested-PCR) (kit, iNtRON, Seongnam-Si, South Korea) targeting the *Orientia tsutsugamushi* 56-kDa type-specific antigen (TSA) gene from the buffy coat or whole blood samples, generating an amplicon of approximately 475 base pairs [16]. The PCR products were verified using 1.2 or 1.5% agarose gel electrophoresis (Seakem LE agarose) and subsequently sequenced to identify the strain type of *Orientia tsutsugamushi* (Genotech, Daejeon, Korea; SolGent, Daegeon, Korea; Macrogen, Seoul, Korea; COSMOGENETECH, Seoul, Korea). The gene sequences were analyzed using the Basic Local Alignment Search Tool (BLAST) against the National Center for Biotechnology Information (NCBI) database to determine the strain pedigree. The obtained sequences (approximately 475 base pairs) were aligned and analyzed using BLAST comparison against reference sequences in the NCBI GenBank database. Strain assignment (Boryong, Taguchi, Karp and Kanda) was made only when the nucleotide identity was ≥ 99% similar with the corresponding reference strain, allowing for high-confidence classification. Cases with ambiguous or low-quality sequences were not assigned to a specific strain and were reported as “not sequenced” or “unclassified”.

#### Diagnosis of hantavirus.

The diagnosis of hantavirus infection was established through nested reverse-transcription polymerase chain reaction (RT-nPCR) and/or a four-fold increase in IgG antibody titers. Viral RNA was extracted from the whole blood or buffy coat (150 µL each) using the Viral Gene-spin Viral DNA/RNA Extraction Kit (iNtRON, Seongnam, Korea) and the QIAamp Viral RNA Mini kit (QIAGEN, Hilden, Germany). RT-nPCR targeted the L segment of hantaviruses, including HTNV and SEOV. Complementary DNA (cDNA) synthesis was performed using SuperScript VILO MasterMix (Invitrogen, Waltham, MA). A positive result was defined as the detection of the viral L segment encoding RNA-dependent RNA polymerase [17]. IgG antibody titers specific to HTNV were measured using an indirect immunofluorescence assay at a commercial laboratory (Green Cross Corp., Yongin, Korea) [17].

### Data analysis

Data were analyzed using STATA 18 (licensed). Categorical data were summarized as frequencies and percentages, while continuous data were summarized as mean ± standard deviation or median and interquartile range (IQR). Comparisons between groups were performed using the chi-squared test and unpaired t-test or Mann-Whitney U test.

For risk factor analysis, those factors with *p* ≤ 0.05 on univariable analysis were included in adjusted analysis with logistic regression to calculate adjusted odds ratio (OR) and 95% confidence interval (CI). Statistical significance was defined as *p* < 0.05.

## Results

A total of 207 patient records were reviewed. Six cases of co-infection with scrub typhus and HTNV were excluded. Of the remaining cases, 156 (77.61%) patients were diagnosed with scrub typhus, and 45 (22.39%) were diagnosed with HFRS.

Among patients with HFRS, 33 (76.74%) tested positive via PCR, and 31 (68.89%) demonstrated a four-fold increase in IgG titers.

Among patients with scrub typhus, the *Orientia tsutsugamushi* strain distribution was as follows: 136 (87.18%) were Boryong strain, 4 (2.56%) were Taguchi strain, 3 (1.93%) were Karp strain, and 1 (0.64%) was Kanda strain. Nine specimens could not be sequenced, and sequencing was not performed for three specimens.

### Sociodemographic, clinical and laboratory parameters

The mean age of patients with scrub typhus was 69.52 ± 11.25 years, with a majority aged ≥65 years (103, 66.03%). In contrast, the mean age of patients with HFRS was 56.84 ± 17.24 years. The detailed sociodemographic characteristics are presented in Table 1.

**Table 1:** Sociodemographic, clinical and laboratory characteristics of patients with scrub typhus infection and hemorrhagic fever with renal syndrome (HFRS) treated at the Chosun University Hospital, South Korea, 2010–2023.

Characteristics	Scrub typhus		Hemorrhagic Fever with Renal Syndrome		*p* value
	n	(%)	n	(%)	
**Sociodemographic parameters**					
Age group (years)					
18 – 24	1	0.64	2	4.44	**<0.001**
25 – 34	0	–	3	6.67	
35 – 44	0	–	4	8.89	
45 – 54	12	7.69	13	28.89	
55 – 64	40	25.64	6	13.33	
≥ 65	103	66.03	17	37.78	
Age (mean ±SD)	69.52	±11.25	56.84	±17.24	**<0.001**
Sex					
Male	61	39.10	29	64.44	**0.003**
Female	95	60.90	16	35.56	
Underlying comorbidities (any)	107	68.59	12	26.67	**<0.001**
Diabetes mellitus	42	26.92	5	11.11	**0.027**
Hypertension	66	42.31	9	20.00	**0.006**
Cerebrovascular accident	8	5.13	1	2.22	0.406
Congestive heart failure	8	5.13	0	–	0.121
Chronic liver disease	4	2.56	0	–	0.278
Chronic kidney disease	2	1.28	0	–	0.445
Chronic obstructive pulmonary disease	2	1.28	0	–	0.445
Solid tumor	13	8.33	0	–	0.405
Farmer	73	46.79	19	42.22	0.588
Month of hospitalization					
March – May	0	–	5	11.11	**<0.001**
June – August	0	–	7	15.56	
September – November	153	98.08	23	51.11	
December – February	3	1.92	10	22.22	
Place of residence					
Province (rural)	133	85.26	36	80.00	0.396
Metropolis (urban)	23	14.74	9	20.00	0.396
**Clinical parameters**					
Fever	143	91.67	23	51.11	**<0.001**
Myalgia	111	71.15	17	37.78	**<0.001**
Fatigue	132	84.62	2	4.44	**<0.001**
Sore throat	40	25.64	1	2.22	**<0.001**
Thirst	90	57.69	1	2.22	**<0.001**
Dyspnea	34	21.79	3	6.67	**0.021**
Gastrointestinal symptoms	120	76.92	20	44.44	**<0.001**
Hemorrhagic symptoms^a^	9	5.77	16	35.56	**<0.001**
Headache	86	55.13	7	15.56	**<0.001**
Altered mental status	25	16.03	5	11.11	**0.415**
Rash	129	82.69	2	4.44	**<0.001**
Eschar	116	74.36	1	2.22	**<0.001**
Course in hospital					
Duration of hospital stay (mean ±SD)	7.89	±7.56	14.09	±7.67	**<0.001**
ICU admission	14	8.97	15	33.33	**<0.001**
Death	5	3.21	1	2.22	0.733
**Laboratory parameters**					
White blood cell (/µL)					
Leukopenia <4000	16	10.26	1	2.22	**<0.001**
Normal (4000 – 11000)	101	64.74	14	31.11	
Leukocytosis >11000	39	25.00	30	66.67	
Median (IQR)	8105	5370, 11035	14840	7940, 21200	<0.001*
Neutrophil <1500/µL	7	4.49	1	2.22	0.494
Neutrophil % (median, IQR)	76.45	69.30, 83.60	66	51.10, 81.30	0.001*
Lymphocyte <1500/µL	148	94.87	15	33.33	<0.001*
Lymphocyte % (median, IQR)	13.00	5.30, 17.50	15.70	9.40, 31.10	0.034*
Hemoglobin (g/dL)					
Normal ≥11	132	84.62	39	86.67	0.734
Hb < 11	24	15.38	6	13.33	
Platelet (/µL)					
Platelet ≥50000	147	94.23	24	53.33	**<0.001**
Platelet <50000	9	5.77	21	46.67	
Median (IQR)	124000	99000, 157000	55000	31000, 124000	<0.001*
Creatinine (mg/dL)^b^					
Normal <2	139	89.10	22	50.00	**<0.001**
Elevated ≥2	17	10.90	22	50.00	
Median (IQR)	0.95	0.76, 1.34	1.93	1.12, 4.80	<0.001*
Proteinuria (mg/g)					
≤ 30	28	18.18	10	22.22	**<0.001**
> 30 – ≤ 100	110	71.43	5	11.11	
> 100 – ≤ 300	16	10.39	3	6.67	
> 300 – ≤ 1000	0	–	15	33.33	
> 1000	0	–	12	26.67	
Bilirubin (mg/dL)					
Normal ≤1.2	128	82.05	40	88.89	0.275
Elevated >1.2	28	17.95	5	11.11	
Aspartate aminotransferase (U/L)					
Normal ≤40	14	8.97	8	17.78	0.096
Elevated >40	142	91.03	37	82.22	
Median (IQR)	90.10	55.85, 139.35	83.40	62.90, 183	0.754
Alanine aminotransferase (U/L)					
Normal ≤40	43	27.56	22	48.89	**0.007**
Elevated >40	113	72.44	23	51.11	
Median (IQR)	63.50	38.10, 108.75	42.80	30.50, 83.30	0.030*
International normalized ratio					
Normal ≤1.3	150	76.77	41	91.11	0.107
Elevated >1.3	5	3.23	4	8.89	
Activated partial thromboplastin time (second)^a^					
Normal ≤35	127	81.94	22	50.00	**<0.001**
Prolonged >35	28	18.06	22	50.00	
C-reactive protein (mg/dL)^b^					
Normal ≤1	2	1.28	4	9.09	**0.007**
Elevated >1	154	98.72	40	90.91	
Median (IQR)	9.60	5.05, 16.5)	7.22	4.08, 11.91	0.019*
Creatine kinase (U/L)^c^					
Normal ≤200	113	75.33	20	47.62	**0.001**
Elevated >200	37	24.67	22	52.38	
Lactate dehydrogenase (U/L)^d^					
Normal ≤300	2	1.44	1	2.94	0.547
Elevated >300	137	98.56	33	97.06	

^a^ Hemorrhatic manifestations included epistaxis, gingival bleeding, hemoptysis, gastrointestinal bleeding, hematemesis/melena, gross hematuria, purpura, petechiae.

^b^ Missing value for one patient in HFRS group.

^c^ Missing value for three patients in HFRS group.

^d^ Missing value of LDH for 11 patients in HFRS group and 17 patients in scrub typhus group.

ICU = intensive care unit; IQR = interquartile range, SD = standard deviation; ULN = upper limit of normal.

*Mann Whitney U Test.

The mean duration of hospitalization was significantly shorter for patients with scrub typhus (7.89 ± 7.56 days) compared to patients with HFRS (14.09 ± 7.67 days; *p* < 0.001). The median hospital stays were 6 days (IQR 4, 8) for patients with scrub typhus and 12 days (IQR 8, 21) for patients with HFRS. A higher proportion of patients with HFRS (15 patients, 33.33%) required intensive care admission compared to those with scrub typhus (14 patients, 8.97%), *p* < 0.001. Eleven patients with scrub typhus requiring ICU admission had Boryong strain, one had Taguchi strain and two did not have sequencing information.

There were 5 deaths (3.21%) among patients with scrub typhus and 1 (2.22%) death among those with HFRS, *p* = 0.733. Three of the deaths among scrub typhus had Boryong strain, the other two did not have sequencing information. The clinical characteristics, hospitalization duration, and outcomes are detailed in Table 1.

**Fig 1:**
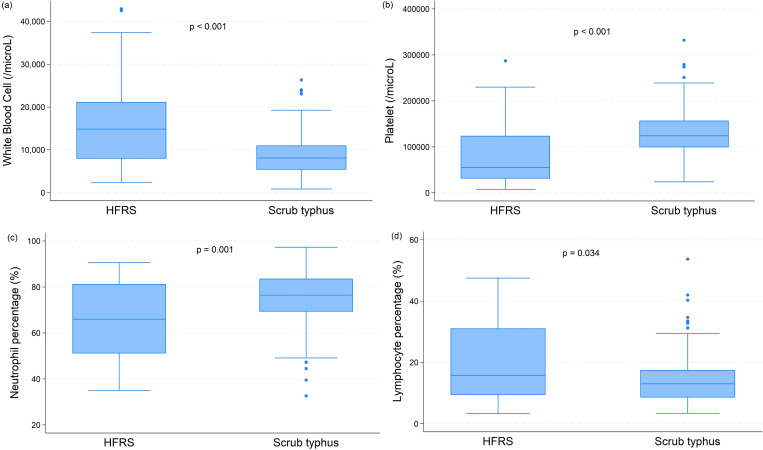
Distribution of (a) leukocyte and (b) platelet counts, and (c) neutrophil and (d) lymphocyte percentages in patients with hemorrhagic fever with renal syndrome (HFRS) and scrub typhus infection treated at the Chosun University Hospital, South Korea, 2010 – 2023.

Patients with scrub typhus had a lower median leukocyte count (8105/µL, IQR 5370, 11035) compared to patients with HFRS (14840/µL, IQR 7940, 21200; *p* < 0.001). Scrub typhus cases had a higher median platelet count (124000/µL, IQR 99000, 157000) compared to HFRS cases (55,000/µL, IQR 31000, 124000; *p* < 0.001). Patients with scrub typhus had a median AST 90.10 U/L (IQR 55.85, 139.35 U/L) while those with HFRS had a median AST 83.40 U/L (IQR 62.90, 183 U/L), *p* = 0.754. Patients with scrub typhus had a median ALT 63.50 U/L (IQR 38.10, 108.75 U/L) while those with HFRS had a median ALT 42.80 U/L (IQR 30.50, 83.30 U/L), *p* = 0.030. Patients with scrub typhus had a median creatinine 0.95 mg/dL (IQR 0.76, 1.34 mg/dL) while those with HFRS had a median creatinine 1.93 mg/dL (IQR 1.12, 4.80 mg/dL), *p* < 0.001. The detailed differences in laboratory parameters between patients with scrub typhus and HFRS are presented in Fig 1 and Table 1.

### Risk factors associated with scrub typhus

The sociodemographic, clinical and laboratory parameters associated with scrub typhus are presented in Table 2. Adjusted analysis identified the following significant associations with scrub typhus: rash (adjusted OR: 1.23, 95% CI: 1.10–1.37, *p* < 0.001), eschar (adjusted OR: 1.16, 95% CI: 1.06–1.26, *p* = 0.001), and lymphopenia (<1500/µL) (adjusted OR: 1.42, 95% CI: 1.16 – 1.74, *p* = 0.001).

**Table 2:** Association of sociodemographic, clinical and laboratory parameters with scrub typhus infection compared to hemorrhagic fever with renal syndrome (HFRS) among patients treated at the Chosun University Hospital, South Korea, 2010–2023.

Risk factors	OR	95% CI	*p* value	Adjusted OR	95% CI	*p* value
Age group (years), Ref 18 – 24						
25 – 34	1					
35 – 44	1					
45 – 54	1.85	0.15 – 23.07	0.634			
55 – 64	13.33	1.04 – 170.63	0.046			
≥ 65	12.11	1.04 – 141.82	0.046			
Sex, Ref Male	2.82	1.42 – 5.63	**0.003**	1.00	0.92 – 1.11	0.914
Farmer	1.20	0.62 – 2.35	0.588			
Month of hospitalization, Ref March – May						
June – August	1					
September – November	22.17	5.68 – 86.62	**<0.001**			
December – February	1					
Place of residence, Ref Metropolis	1.45	0.62 – 3.40	0.398			
Clinical parameters						
Fever	10.52	4.66 – 23.77	**<0.001**	1.06	0.92 – 1.21	0.433
Myalgia	4.06	2.03 – 8.14	**<0.001**	1.04	0.96 – 1.13	0.362
Sore throat	15.17	2.02 – 113.74	**0.008**	1.05	0.96 – 1.15	0.266
Thirst	60.00	8.06 – 446.63	**<0.001**	1.01	0.93 – 1.09	0.801
Dyspnea	3.90	1.14 – 13.37	**0.030**	1.00	0.93 – 1.10	0.861
Gastrointestinal symptoms	4.17	2.08 – 8.36	**<0.001**	1.02	0.93 – 1.11	0.707
Hemorrhagic symptoms	0.11	0.04 – 0.28	**<0.001**	0.90	0.77 – 1.05	0.191
Headache	6.67	2.80 – 15.85	**<0.001**	1.04	0.96 – 1.12	0.347
Altered mental status	1.53	0.54 – 4.25	0.418			
Rash	102.72	23.45 – 449.99	**<0.001**	**1.23**	**1.10 – 1.37**	**<0.001**
Eschar	127.60	17.02 – 965.49	**<0.001**	**1.16**	**1.06 – 1.26**	**0.001**
Duration of hospital stay	0.91	0.87 – 0.94	**<0.001**			
Death	1.46	0.17 – 12.80	0.734			
Laboratory parameters						
White blood cell (/µL), Ref 4000 – 11000						
Leukopenia <4000	2.21	0.27 – 18.04	0.456	1.04	0.92 – 1.17	0.535
Leukocytosis >11000	0.18	0.09 – 0.38	**<0.001**	0.97	0.89 – 1.06	0.455
Neutrophil <1500/µL	2.07	0.25 – 17.26	0.502			
Lymphocyte <1500/µL	37.00	14.40 – 95.06	**<0.001**	**1.42**	**1.16 – 1.74**	**0.001**
Hemoglobin (g/dL), Ref ≥ 11	1.18	0.45 – 3.10	0.734			
Platelet (/µL), Ref ≥ 50000	0.07	0.03 – 017	<0.001	1.06	0.89 – 1.24	0.524
Elevated creatinine (mg/dL), Ref < 2	0.12	0.06 – 0.27	**<0.001**	1.02	0.90 – 1.15	0791
Proteinuria (mg/g), Ref ≤ 30						
> 30 – ≤ 100	7.85	2.48 – 8.33	**<0.001**	1.10	0.99 – 1.21	0.071
> 100 – ≤ 300	1.90	0.45 – 7.95	0.377	1.09	0.94 – 1.25	0.244
Bilirubin (mg/dL), Ref ≤ 1.2	1.75	0.63 – 4.83	0.280			
Aspartate aminotransferase (U/L), Ref ≤ 40	2.19	0.85 – 5.62	0.102			
Alanine aminotransferase (U/L), Ref ≤ 40	2.51	1.27 – 4.97	**0.008**	1.03	0.94 – 1.11	0.559
International normalized ratio, Ref ≤ 1.3	0.34	0.08 – 1.33	0.122			
Activated partial thromboplastin time (second), Ref ≤ 35	0.22	0.11 – 0.45	**<0.001**	1.02	0.93 – 1.12	0.720
C-reactive protein (mg/dL), Ref ≤ 1	7.70	1.36 – 43.55	**0.021**	1.52	0.74 – 3.13	0.257
Creatine kinase (U/L), Ref ≤ 200	0.30	0.15 – 0.61	**0.001**	0.94	0.86 – 1.02	0.158
Lactate dehydrogenase (U/L), Ref ≤ 300	2.08	0.18 – 23.59	0.556			

## Discussion

Scrub typhus and HFRS present as acute febrile illnesses with overlapping but non-specific symptoms sometimes leading to misdiagnosis if not supported by reliable laboratory evidence [18]. In this study, the highest proportion of both scrub typhus and HFRS was observed in individuals aged ≥65 years. In a review of scrub typhus cases from 2001 to 2013 in South Korea, the highest incidence was reported in the 60–69-year age group [4]. This contrasts with a median age of 33 years reported in Vietnam [19] and 48 years in South India [20]. For HFRS, an earlier study in South Korea (2002–2012) found the highest proportion of cases in individuals aged >40 years [21]. In China, the at-risk age group for HTNV was 40–59 years [22]. In the United States, HCPS cases confirmed through serology or PCR, PUUV, SEOV, Prospect Hill virus, and SNV showed a median age of 34.9 years [23]. Similarly, HCPS caused by Andes virus in Argentina (1995–2008) predominantly affected individuals aged 21–30 years [24], while cases in Chile were reported in the 15–44-year age group [25].

In South Korea, individuals aged ≥65 years represent the main workforce engaged in outdoor and agricultural activities, particularly during the harvest season between October and December when exposure to chigger mites (*Leptotrombidium* spp.) and infected rodents is highest [26]. This demographic pattern reflects socio-economic and demographic shifts, as younger populations have increasingly migrated to urban areas, leaving older adults to perform farming and fieldwork in rural communities [6,27,28]. Consequently, older adults experience greater exposure to vector habitats, leading to a higher incidence of both *Orientia tsutsugamushi* (scrub typhus) and HTNV/SEOV infections. Additionally, the rodent population density and virus shedding rates rise after the breeding season in late summer and early autumn, further elevating the risk of human infection [11]. These findings are consistent with previous national epidemiological data showing that most scrub typhus and HFRS cases in Korea occur among elderly farmers during the autumn season [27,28].

A higher proportion of scrub typhus cases occurred in females. A global review of epidemiological studies identified females as an at-risk population for scrub typhus [3], with slightly higher disability-adjusted life years compared to males [29]. Conversely, HFRS was more prevalent among males, consistent with a reported male-to-female ratio of 2.6:1 in adults, while the ratio was 1:1 among children [9]. The observed sex and age distributions in scrub typhus and HFRS may be explained by differences in human activities, occupational exposure, and season-related risk-factors.

Fever is a hallmark symptom of scrub typhus [5,30], reported in nine out of ten patients in this study. While a febrile phase lasting 4–6 days is typically described in HFRS [31], only half of the patients in our study reported fever, in contrast to the 86.9-97.1% reported in studies from Korea and China [22,32] and 100% in critical ill cases [33]. In cases with high-risk exposure to rodent carriers, Hantavirus infection should be considered a differential diagnosis even in the absence of fever. Severe cases of HTNV or Dobrava virus (DOBV) infection typically exhibit five clinical phases, but these may not be apparent in less severe forms caused by PUUV or SEOV [11,34]. Prospective studies with detailed clinical descriptions are recommended to characterize variant-specific symptoms of Hantavirus infections.

In scrub typhus, diffuse, macular or maculopapular rashes appear 3–10 days after fever onset [30], while eschars may develop up to one week before fever [35]. In our study, patients with scrub typhus were more likely to have skin rashes and eschars compared to those with HFRS. Eschars may be a diagnostic clue but may not always be present. The proportion of eschars observed in this study was consistent with reports of 78.9-80.4% in Korea [5,36]. However, eschars have been reported globally in only 30.3% of cases, with lower rates in South Asia [37]. Despite variability, head-to-toe clinical examinations, including areas under compression or restrictive clothing, remain essential [38].

Higher proportions of hemorrhagic symptoms and severe thrombocytopenia (platelets <50,000/µL) were observed in HFRS cases. Bleeding manifestations included petechiae, epistaxis, menorrhagia, and gastrointestinal bleeding. While fatal bleeding is rare, it is more common in HTNV and DOBV infections [9]. The primary mechanisms of hemorrhage in HFRS are increased vascular permeability and thrombocytopenia, which also contribute to renal impairment [11]. Renal injury results from tubular damage caused by cell infiltration and increased glomerular permeability, leading to massive proteinuria [11].

In our study, leukocytosis was more frequently observed in HFRS cases compared to cases with scrub typhus. Hantavirus infections are associated with elevated leukocyte populations [39,40] and a marked increase in activated T cells, which contribute to cytokine storms [41]. This immune cascade, involving natural killer and T cell activation, correlates with viremia and may offer targets for therapeutic interventions [42].

Conversely, in our study, scrub typhus was associated with lymphocytopenia (<1500/µL) during the acute phase, with counts returning to baseline during convalescence [43]. This phenomenon may result from CD4+ and CD8+ T cell apoptosis during the acute phase, followed by CD8+ T cell proliferation in the convalescent phase. In hantavirus infections, increased percentages of CD8+ T cells with activation markers (e.g., CD71 and CD25) correlate positively with AST and ALT levels and a negatively with platelet counts [44]. Further research is warranted to elucidate these immune mechanisms.

Scrub typhus can cause multiorgan dysfunction, including respiratory distress, acute kidney injury, and meningoencephalitis [30]. Bleeding manifestations occur in up to 6.2% of scrub typhus cases [37]. Disease severity is influenced by host factors, pathogen factors (e.g., *Orientia tsutsugamushi* strain) [45], and bacterial load in specific organs [46]. Strains such as Karp, Gilliam, and Woods have been associated with higher levels of endothelial cell activation markers [46], which may explain variations in clinical severity. Given the overlapping clinical features of scrub typhus and HFRS, further studies are required to understand their full clinical spectrum.

In our study, patients with HFRS had relatively longer hospital stays and higher rates of ICU admission compared to those with scrub typhus. The duration of hospitalization for scrub typhus was consistent with reports from other countries [20]. In a review of 114 patients with HFRS, the average duration of hospital stay was 10.98 ± 4.81 days among non-pregnant female patients and 11.15 ± 4.41 days among pregnant patients [47]. In another review of 35 patients with HFRS, the average duration of hospital stage was 11.7 ± 5.3 days [32]. Patients at our hospital had a slightly longer stay in hospital that may reflect disease severity, delays in diagnosis, or low prioritization of HTNV testing.

This study was based on a retrospective review of data from a single teaching hospital. Comparisons of clinical and laboratory parameters are conducted only for PCR-confirmed cases. While a few cases might have been missed in instances where tests were not offered, the number might be negligible given the relatively easy access to molecular testing for diagnosis. Given that scrub typhus and HFRS have overlapping clinical findings and that only a small number of cases with HFRS were captured in this study, further prospective multicenter studies are necessary to validate the differences in clinical and laboratory parameters presented here.

## Conclusions

In our study, patients with scrub typhus and HFRS presented with overlapping clinical features and had similar mortality rates. However, patients with skin rash, eschar and lymphopenia were more likely to have scrub typhus. The duration of hospital stay was longer in patients with HFRS. Further studies are recommended to describe the clinical features and disease course in patients with various subtypes of Hantavirus infections.

## Supporting information

S1 Data Contains the complete anonymized dataset. (XLSX)

S1 File Summary. Shows the total number of patients, data variables collected and the key findings.
(TIF)
