# Supplementary figures and images for: Clinical and laboratory factors associated with scrub typhus and hemorrhagic fever with renal syndrome in Southwestern Korea: A cross-sectional study
Source: PLoS Negl Trop Dis. 2025 Nov 7;19(11):e0013709. doi: 10.1371/journal.pntd.0013709 (PMC12629494; doi:10.1371/journal.pntd.0013709)

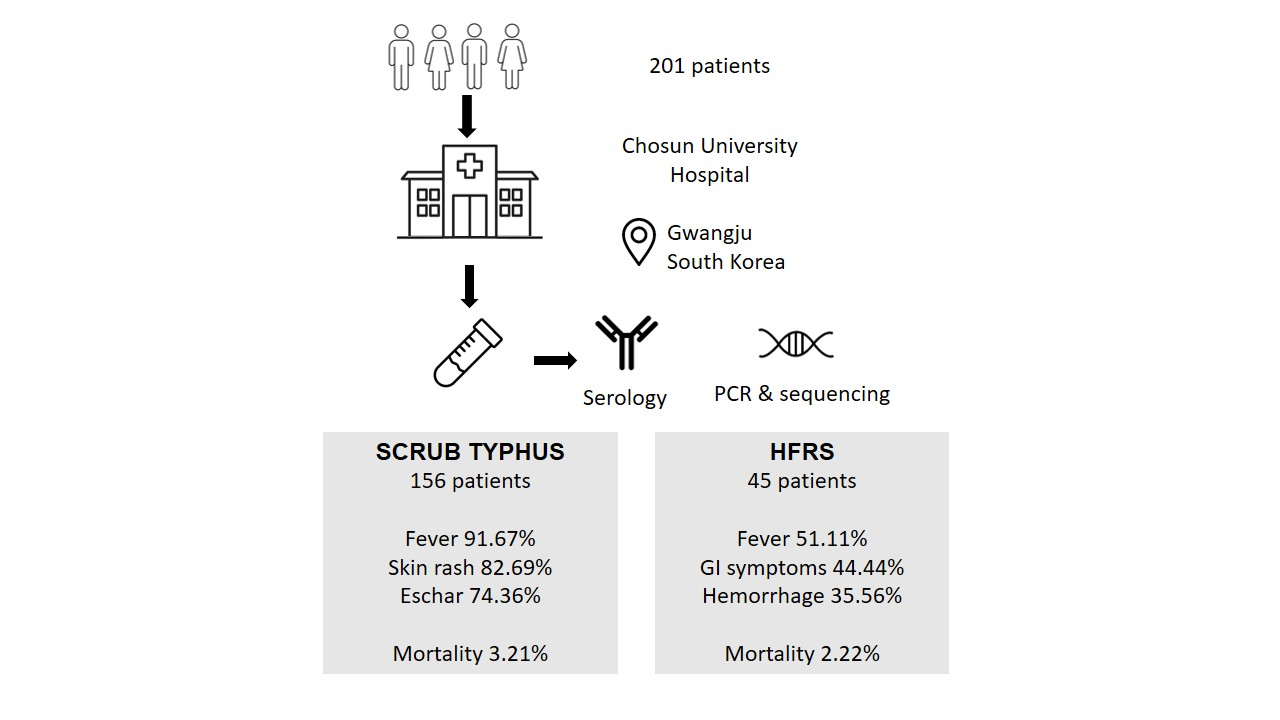

Supplement: S1 File — Shows the total number of patients, data variables collected and the key findings. (TIF) [file pntd.0013709.s002.tif]
